# The Role of Pathology-Based Methods in Qualitative and Quantitative Approaches to Cancer Immunotherapy

**DOI:** 10.3390/cancers14153833

**Published:** 2022-08-08

**Authors:** Olga Kuczkiewicz-Siemion, Kamil Sokół, Beata Puton, Aneta Borkowska, Anna Szumera-Ciećkiewicz

**Affiliations:** 1 Department of Pathology, Maria Sklodowska-Curie National Research Institute of Oncology, 02-781 Warsaw, Poland; 2 Diagnostic Hematology Department, Institute of Hematology and Transfusion Medicine, 02-776 Warsaw, Poland; 3 Department of Soft Tissue/Bone Sarcoma and Melanoma, Maria Sklodowska-Curie National Research Institute of Oncology, 02-781 Warsaw, Poland

**Keywords:** immune profiling, multiplex, image analysis, spectral imaging, digital pathology, artificial intelligence, PD-L1

## Abstract

**Simple Summary:**

Immunotherapy has become the filar of modern oncological treatment, and programmed death-ligand 1 expression is one of the primary immune markers assessed by pathologists. However, there are still some issues concerning the evaluation of the marker and limited information about the interaction between the tumour and associated immune cells. Recent studies have focused on cancer immunology to try to understand the complex tumour microenvironment, and multiplex imaging methods are more widely used for this purpose. The presented article aims to provide an overall review of a different multiplex in situ method using spectral imaging, supported by automated image-acquisition and software-assisted marker visualisation and interpretation. Multiplex imaging methods could improve the current understanding of complex tumour-microenvironment immunology and could probably help to better match patients to appropriate treatment regimens.

**Abstract:**

Immune checkpoint inhibitors, including those concerning programmed cell death 1 (PD-1) and its ligand (PD-L1), have revolutionised the cancer therapy approach in the past decade. However, not all patients benefit from immunotherapy equally. The prediction of patient response to this type of therapy is mainly based on conventional immunohistochemistry, which is limited by intraobserver variability, semiquantitative assessment, or single-marker-per-slide evaluation. Multiplex imaging techniques and digital image analysis are powerful tools that could overcome some issues concerning tumour-microenvironment studies. This novel approach to biomarker assessment offers a better understanding of the complicated interactions between tumour cells and their environment. Multiplex labelling enables the detection of multiple markers simultaneously and the exploration of their spatial organisation. Evaluating a variety of immune cell phenotypes and differentiating their subpopulations is possible while preserving tissue histology in most cases. Multiplexing supported by digital pathology could allow pathologists to visualise and understand every cell in a single tissue slide and provide meaning in a complex tumour-microenvironment contexture. This review aims to provide an overview of the different multiplex imaging methods and their application in PD-L1 biomarker assessment. Moreover, we discuss digital imaging techniques, with a focus on slide scanners and software.

## 1. Introduction

Immunotherapy revolutionises the paradigm of cancer treatment. The concept is based on blocking immune-checkpoint proteins such as programmed cell death protein 1 (PD-1), programmed death-ligand 1 (PD-L1), or cytotoxic T-lymphocyte-associated antigen 4 (CTLA-4), so that the anti-tumour T-cell response remains active [1]. Nowadays, this approach is proven experimentally and translated to clinical practice. Immunotherapy based on the inhibition of the PD-1/PD-L1 axis is the most widely implicated in the clinic. Several immune-checkpoint blockade drugs have been approved by the Food and Drug Administration (FDA) and European Medicines Agency (EMA) to treat a diverse range of malignancies, including Hodgkin lymphoma, melanoma, Merkel cell carcinoma, and non-small-cell lung carcinoma (NSCLC), breast, renal, bladder, head and neck, gastric and hepatocellular carcinoma [2,3,4,5,6,7,8].

Before starting the anti-PD-L1 treatment, in most the cases, the evaluation of PD-L1 expression in tumour and/or immune cells is demanded, as this correlates with a positive response [9]. Different methods could assess the PD-1/PD-L1 axis, but evaluation at the tissue level seems to be the most critical qualification for targeted therapy. The pathological techniques are mainly based on formalin-fixed paraffin-embedded (FFPE) samples used in routine diagnostics. To assess the status and level of expression, a variety of companion immunohistochemical assays are approved with different scoring systems, antibodies, and platforms. Each drug has its own protocol of PD-L1 measurement and different expression cut-offs qualifying it for therapy [9,10,11]. Three main scoring systems adopted for PD-L1 assessment are: the tumour proportion score (TPS), a calculation of the proportion of positive tumour cells; the combined positive score (CPS), defined by the ratio of total positive tumour and immune cells to the total number of viable tumour cells; and the percentage of PD-L1 positive tumour-infiltrating immune cells [12,13]. However, despite defined criteria, the assessment is semiquantitative and test interpretation is subjective. When comparing different scoring systems, the TPS is much easier to interpret than CPS, probably due to immune cell assessment in the latter [14]. In addition, the need to evaluate PD-L1 expression using a specific diagnostic assay for a given drug is unreasonable and causes disarray during analysis of the results by pathologists or oncologists. A range of harmonisation studies of PD-L1 assays were performed, showing that while the estimation of PD-L1 expression in tumour cells was consistent among pathologists, the concordance in the assessment of PD-L1 in immune cells was poor [14,15,16,17,18,19,20,21]. This raises questions on the accuracy and reproducibility of PD-L1 evaluation in standard chromogenic immunohistochemistry (IHC).

Looking further ahead, the assessment of PD-L1 expression is facing other issues. Doubts concerning the true predictive value of this marker are rising as knowledge about the relationship between a tumour and its microenvironment evolves. PD-L1 distribution exhibits topographic intratumour heterogeneity and fluctuates with tumour growth [22]. Moreover, the administration of any therapy, including immunotherapy itself, changes the interactions of the PD-1/PD-L1 axis [23,24].

Patient-centred oncological care requires long-distance decisions, and sequential therapies are becoming standard in cancer treatment approaches. The evaluation of the spatial distribution and density of immune cells, together with PD-L1 expression, may lead to more informed choices for therapy directions, especially while introducing immune-checkpoint inhibitors [22]. Diagnostic accuracy in PD-L1 expression and immune profiling could be improved by introducing diagnostic methods based on multiplexing techniques [25,26].

Up-to-date semiquantitative evaluation of PD-L1 expression by pathologists needs to be supported by more objective tools, including the handling of virtual slides and quantitative approaches using digital machinery. Here, we review various methods of multiplexed imaging, covering its fundamentals, advantages, and limitations.

## 2. Multiplex Immunohistochemistry/Immunofluorescence (mIHC/IF) Techniques

**Figure 1:**
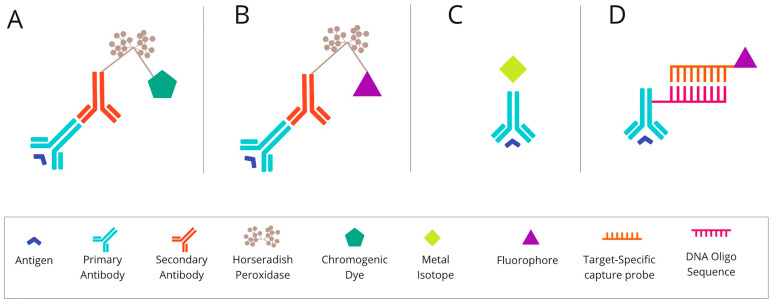
The basic mechanism of each of the mIHC/IF techniques. (**A**) Chromogenic-based: antigen-specific primary antibody is bound to a secondary antibody, conjugated with HRP enzymes labelled with chromogen. (**B**) Fluorescence-based: antigen-specific primary antibody is attached to a secondary antibody conjugated with HRP enzymes. Detection is achieved using the fluorophore-labelled HRP substrate, tyramide. (**C**) Metal-based: a primary antibody bound to the target antigen is labelled with an isotopically pure metal-chelator tag. (**D**) DNA-barcoding-based: a primary antibody bound to the target antigen is labelled with a unique DNA oligonucleotide tag. Subsequently, a complementary strand of DNA coupled to a specific fluorophore is attached. Abbreviations: mIHC/IF—multiplex immunohistochemistry/immunofluorescence; HRP—horseradish peroxidase; IHC—immunohistochemistry.

The mIHC/IF techniques can be divided into (i) chromogenic-based, (ii) fluorescence-based, (iii) metal-based, and (iv) DNA-barcoding-based methods [27]. The principles of each method are described graphically in Figure 1 below.

### 2.1. Chromogenic-Based mIHC Is Widely Based on Immunohistochemical (Standard Single-Antibody Chromogenic Immunohistochemistry) Technologies

One of the most common diagnostic methods is the standard IHC. The IHC technique detects cell proteins using specific antibodies as a result of the antigen–antibody reaction. The necessary steps to perform IHC staining are as follows. Fixed tissue samples are embedded in paraffin, or rarely, frozen when there is no other possibility. The biopsies are thinly sliced (3–5 µm), placed on glass slides, and dried. Subsequently, the antigen discovery procedure is performed to improve protein identification by the antibody. The general purposes of epitope retrieval are the denaturation of proteins, the removal of methylene bridges, and the minimisation of chemical forces, which may interfere with the antigen measured. However, the methods of epitope recovery rely mainly on delivering energy to the tissue in the process of heating in a buffer, commonly based on citrate, or on high- or low-pH liquid. The next stage is to prevent non-specific staining by carrying out two blocking steps based on either horseradish peroxidase (HRP) or serum-free protein [28,29].

**Figure 2:**
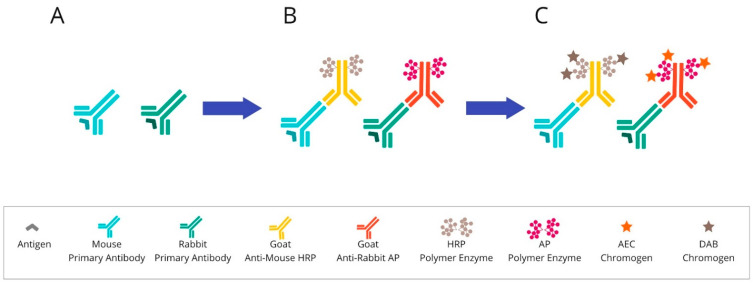
Overview of chromogenic-based mIHC: (**A**) After primary antibody incubation, (**B**) secondary antibodies labelled with polymer enzymes are conjugated. (**C**) The HRP or AP are reacted with an appropriate substrate bound to a chromogenic dye, such as DAB or AEC, leading to the precipitation of insoluble, coloured products. Abbreviations: HRP—horseradish peroxidase; AP—alkaline phosphatase; DAB—3,3′-diaminobenzidine; AEC—3-Amino-9-ethylcarbazole.

Once the blocking step has occurred, the sample is incubated with the proper antibody. The antibody should be at the correct concentration in order to retain the specificity of the staining without any background interference. Then, the tissue material is incubated with the secondary antibody to visualise the binding to the primary antibody. These systems are most often based on anti-mouse or anti-rabbit polymers, which are conjugated by HRP enzymes. The detection system in chromogenic tests relies on the colour reaction of the enzyme-labelled complex with the chromogen. For these assays, the chromogen is 3,3′-diaminobenzidine, the oxidation of which causes the formation of a brown-coloured product. Another widely used chromogen is 3-Amino-9-ethylcarbazole (AEC). It appears as a red colour on a tissue and becomes discoloured in organic solvents. The final steps concern the procedure of counterstaining, which can bring out other structures in the coverslip application [30,31]. An overview of chromogenic-based mIHC is presented in Figure 2.

Furthermore, many new chromogens have recently been discovered. Due to their different colours, this discovery made it possible to perform multiplexing using chromogenic IHC. This technique is advantageous in cases where the markers are not located in the same place in the tissue. Interestingly, it is sometimes possible to use multiple chromogens to stain one site simultaneously. Chromogen’s overlapping and characteristic colour change enable the recognition of biomarker co-expression. The multiplex chromogenic IHC technique allows the marking of about 3–5 markers on a whole single section over 10–15 h [32,33].

Because multiplex chromogenic IHC is based on immunohistochemistry technology, it is easy to introduce and apply in tumour-microenvironment studies and in describing the PD-1/PD-L1 pathway.

Chromogenic-based mIHC was used for the determination of the clinicopathological significance of PD-1, LAG3, and TIM3 markers in stage II/III gastric cancer patients [34] and for the identification the phenotypes and spatial profiles of intratumoural PD-1+ helper T cells associated with the prognosis of head and neck squamous cell carcinoma (HNSCC) [35]. Some analytical validation attempts of automated multiplex chromogenic IHC for diagnostic and predictive purposes could potentially be made in the clinic for NSCLC patient care. Ilié et al. presented two validated multiplex chromogenic IHC assays, including TTF1, p40, PD-L1, CD8, ALK, ROS1, BRAF V600E, and TRK, which could be adopted in most laboratories [36].

**Multiplexed immunohistochemical consecutive staining on a single slide (MICSSS)** is performed on FFPE tissue samples, and it relies on multiple cycles of staining with various antibodies, scanning, and destaining of a chromogenic substrate. Each cycle of these methods begins with incubation in a pH-optimised buffer to reveal epitopes. Notably, the MICSSS staining protocol must include an organic solvent-soluble chromogen to become easily removable between cycles. The 3-Amino-9-ethylcarbazole (AEC) is, by far, the most popular MICSSS chromogen. At the end of each subsequent cycle, the epitope retrieval is repeated as soon as chemical destaining is performed. An additional crucial protocol stage includes the blocking of the previous primary antibody. According to the literature, glycine-sodium dodecyl sulphate (SDS), potassium permanganate (KMnO4), and Fab-blocking antibodies are being used for these purposes. The preferred method should be Fab-fragment blocking with antibodies directed against primary antibodies, but from a different species. This action prevents staining interference with previous immunostaining cycles [33,37,38].

Like a simple chromogenic-based mIHC technique, MICSS is broadly used in immuno-oncology research. MICSSS panels can include immune markers that stain different cell compartments. Contrary to other mIHC techniques, MICSSS has an effective system of destaining and blocking between cycles, so steric hindrance is not an issue for this method. For example, it allows for routine membranous staining for CD2, CD3, CD8, and PD-1 quadruple-positive T cells, or the cytoplasmic expression of HLA-DR, CD206, CD68, CD163 quadruple-positive histiocytes without obvious steric hindrance [38,39]. In fact, the method is still useful for tumour-microenvironment studies. A recent paper presented at the 2022 American Society of Clinical Oncology (ASCO) Annual Meeting has described MICSS analysis of the immune microenvironment of bile duct cancers in tumour samples before and after neoadjuvant chemotherapy [40].

### 2.2. Fluorescence-Based mIHC/IF Is a Method That Provides Simultaneous Detection of Multiple Fluorescently Tagged Proteins of Interest in FFPE Tissue Sections

The approaches to fluorescent multiplexing include: (i) direct immunofluorescence: multiple antigen-specific primary antibodies conjugating at distinct fluorophores are involved in this approach. The sensitivity for low-abundance targets is limited due to a lack of signal amplification. Only a subset of protein targets (with the highest cellular concentration) are reliably detected with subsequent imaging; (ii) indirect immunofluorescence: the antigen is detected by fluorescently labelled secondary antibodies specific to the host species in which each primary antibody was raised. The disadvantage of this approach is the limited number of available host species. Multiplexing techniques allow for the use only of a combination of primary antibodies from distinct animal species [41]; and (iii) deposition assays: enzyme-labelled antibodies and tyramide–fluorophore conjugates are involved in this approach [16].

An example of an mIHC/IF method involving tyramide–fluorophore conjugates is **tyramide signal amplification (TSA)**. This approach first requires the application of a primary antibody specific to the protein of interest and a primary specific secondary antibody conjugated to HRP. Detection is achieved using the fluorophore-labelled HRP substrate, tyramide [42]. HRP converts tyramide into a highly reactive oxidised intermediate that binds covalently to tyrosine residues on or near the protein of interest. Because the primary/secondary antibody pair can be removed from the sample without disrupting the antigen-associated fluorescence signal, multiple rounds of staining can be performed [43]. Subsequent visualisation of the amplified signal is enabled by the direct imaging of fluorophores or the downstream application of individual fluorophores tagged to anti-DNP or anti-biotin particles [44].

**Figure 3:**
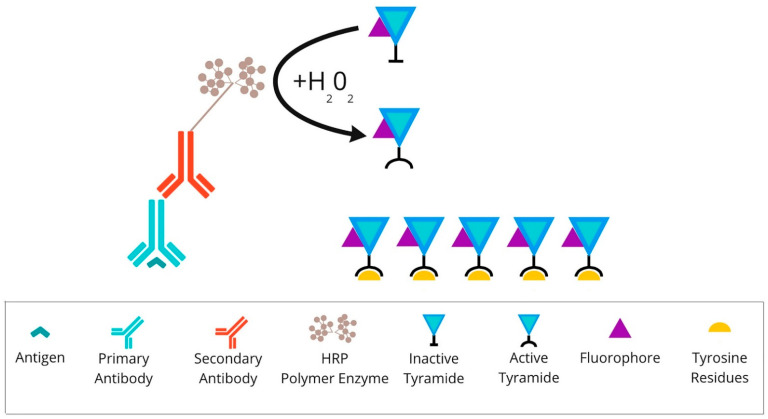
Overview of tyramide signal amplification technique: After primary antibody incubation, secondary antibody labelled with HRP polymer enzymes is conjugated. Detection is achieved using the fluorophore-labelled HRP substrate, tyramide. HRP converts tyramide into a highly reactive oxidised intermediate that binds covalently to tyrosine residues present on or near the protein of interest. Abbreviations: HRP—horseradish peroxidase.

An example of a system which reflects the principle of TSA (presented in Figure 3) is the Opal mIHC assay by Akoya Biosciences (Marlborough, MA, USA). In this method enzymatic amplification is obtained by binding the fluorescent dyes with tyramide molecules. The technique utilises conventional fixation of the primary antibody on the epitope of interest. Next, the secondary antibody binds to the primary one, followed by the HRP polymer enzyme deposition. The HRP converts tyramide into a highly reactive oxidised intermediate that adheres covalently to tyrosine residues present in the area of the protein of interest. This detection is possible due to the labelling of the tyramide via fluorophore. This run can be repeated at least 7–9 times. The TSA multiplex technique allows the staining of multiple biomarkers within a single paraffin slide at the same time. To perform another round of staining, used antibodies need to be stripped via specific microwave treatment. This approach decreases the tissue autofluorescence for each antibody cycle [45,46].

Multiplex immunofluorescence, including the TSA method, enables the simultaneous detection of multiple markers on an individual tissue section. This method has gained importance as a method of immune profiling the tumour microenvironment or identifying targetable biomarkers, such as PD-L1, for studying the effect of immunotherapy. Recently, numerous diverse panels to analyse the tumour microenvironment for patterns of prognostic value have been created [47,48,49,50,51].

The protocol design depends on the research hypothesis, the cell population of interest, or the investigated therapy. The approach based on individual markers recognises a broad number of cell phenotypes, including rare cells, which may be helpful in tumour-microenvironment studies [24]. The TSA method is becoming an essential tool for characterising the tumour microenvironment of various cancers such as Hodgkin lymphoma [52], breast cancer [53], and NSCLC [54].

Parra et al. extensively review the basic requirements for performing TSA in FFPE cancer tissues to support translational oncology research. They stained approximately 4000 tumour samples for the immunoprofiling of labelled biomarkers in a single slide to explain cancer biology at the protein level and identify therapeutic targets and biomarkers [55].

### 2.3. Metal-Based mIHC/IF Are Methods That Utilise Antibodies Conjugated with Isotopically Pure Metal-Chelator Tags

A sample analysis is made using a mass cytometer, which distinguishes signals based on diverse atomic mass [56]. Examples of techniques grounded in the metal-based multiplexing methodology include imaging mass cytometry (IMC) and multiplexed ion-beam imaging (MIBI) [57].

**Figure 4:**
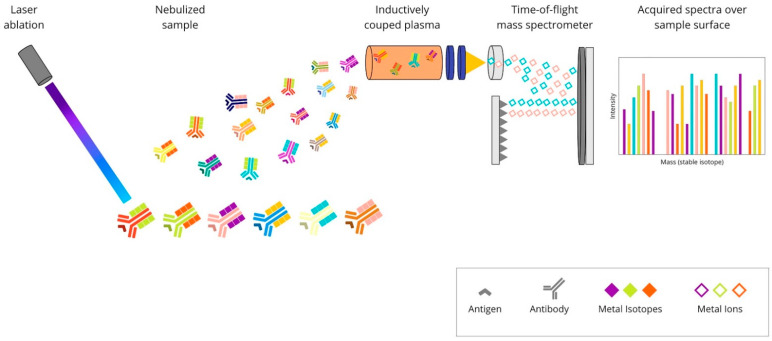
Overview of imaging mass cytometry technique: A mixture of antibodies is labelled with isotopically pure metal-chelator tags. Each antibody binds to a single protein target. Then, the sample is ablated using a laser beam. It generates the separation of particles, which are then carried by a helium/argon mixture stream into a time-of-flight mass spectrometer, where metal ions are separated based on mass. The measured reporter signals are then mapped using the coordinates of each laser spot, and finally, an image is generated based on these data.

**Imaging mass cytometry (IMC)** is a technology which combines laser ablation and mass cytometry by time-of-flight (CyTOF). It empowers target detection via metal-tag labelling. The analysis of up to 100 markers on a single tissue section is possible [58]. What is important is that the data on cellular morphology and tissue architecture are maintained [59]. The tissue sections are stained with antibodies of interest, and standard immunostaining procedures are applied. First, the antigen-retrieval buffer is added to expose the antigen epitope. After that, the sample is incubated with a mixture of antibodies conjuncted with metal tags. Subsequently, regions of interest (ROIs) are selected and recorded by a camera integrated with the Hyperion Tissue Imager, which is part of Hyperion Imaging System (Fluidigm Canada Inc., Markham, Ontario, Canada). A key step in the process is tissue slide-ablation using a laser pulse. The vaporisation of a sample provokes the release of particles, which are then transferred to the CyTOF detector by a stream of inert gas. The measured reporter signals are then profiled using the correspondent of a distinct laser spot. At the end, an image is generated based on these data [60]. The principles of the IMC method are presented in Figure 4.

Ijsselsteijn et al. created a panel of 40 markers, including PD-1 and PD-L1, for imaging mass cytometry of FFPE tissues, with a focus on cancer immunology [61]. IMC has found an application in determining the tumour microenvironment of patients with melanoma [62], oral squamous cell carcinoma [63], cutaneous squamous cell carcinoma [59], or classic Hodgkin lymphoma [64]. The technology used by Elaldi et al. allows the characterisation of the overall tumour organisation, not only that of immune cells. The panel contains markers of epithelial tumour cells, structural markers (blood and lymphatic vessels, nerve fibres, fibroblasts, and extracellular matrix proteins), and many markers for immune cells, including PD-1 and PD-L1 [59].

**Figure 5:**
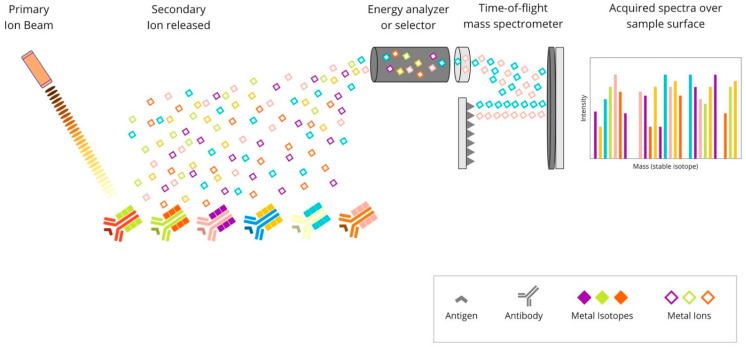
Overview of multiplexed ion-beam imaging technique: Mixture of antibodies are labelled with isotopically pure metal-chelator tags. Each antibody binds to a single protein target. Then, a thin layer of the sample surface is ablated using an oxygen-based primary ion beam. Metal isotopes are released from antibodies as secondary ions, which are then transported to a time-of-flight mass spectrometer. Each unique metal ion represents a protein.

**The multiplexed ion-beam imaging (MIBI)** methodology utilises metal-chelator tags attached to antibodies as in the previously described IMC technique. The difference is in the sample analysis, which in the MIBI technique is based on time-of-flight secondary-ion mass spectrometry (ToF-SIMS). A tissue section is ablated using an oxygen primary-ion beam, which results in the release of metal isotopes from antibodies as secondary ions. Then, the liberated particles are transported to a time-of-flight mass spectrometer, which assigns them to distinct targets based on their atomic-mass detection. Each unique metal ion represents a protein. The created image is high-dimensional and shows the expression of numerous proteins. Due to its high sensitivity and imaging resolution, ToF-SIMS seems to be particularly suitable for tissue imaging [65]. The principles of this technique are presented in Figure 5.

The MIBI technique was successfully used for generating a proteomic profile of a triple-negative breast cancer (TNBC) microenvironment using a panel of 36 markers. Angelo’s group revealed variability in the composition of tumour-immune populations across 41 patients with TNBC. Subsequently, spatial analyses identified tumours that were either immune “mixed” or “compartmentalised” concerning PD-1, PD-L1, and IDO expression patterns. The researchers found a correlation between a quantifiable defined “compartmentalised” architecture and improved overall survival [66].

### 2.4. DNA Barcoding-Based mIHC/IF Are Techniques for Multiplexed Imaging Based on DNA Barcoding Using Oligonucleotide Detection Technologies

**Figure 6:**
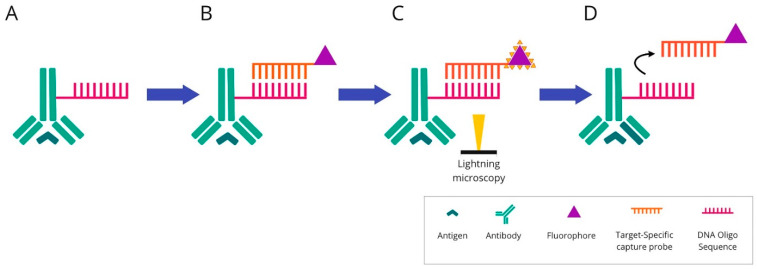
Overview of COdetection by indEXing technique: (**A**) Mixture of target antibodies conjugated with unique oligonucleotide barcodes are used simultaneously to stain a tissue section. (**B**) Then, fluorescently labelled complementary oligonucleotides are added. (**C**) The visualisation is conducted via light microscopy. The targets are detected and imaged in cycles of three targets in each cycle. (**D**) After imaging, the reporter oligonucleotides are stripped using a stripping buffer. The cycle is repeated until all antibodies within the panel have been revealed and visualised.

**Figure 7:**
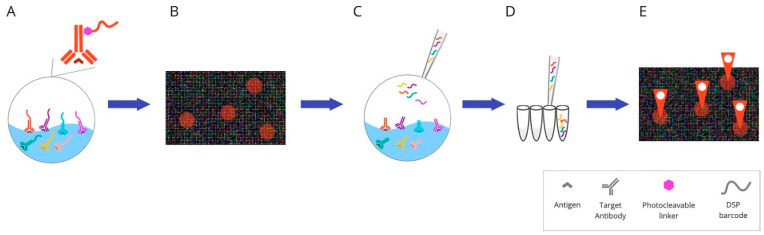
Overview of digital spatial-profiling technique: (**A**) Mixture of target antibodies conjugated with unique oligonucleotide barcodes through a UV photocleavable linker. (**B**) The oligonucleotide barcodes undergo quantitative analysis and are mapped back to tissue location to allow spatial profiling at the defined ROIs. (**C**) Sequential UV laser light exposure of each ROI results in the sample’s release of indexing oligonucleotide tags. (**D**) Then, a small pipette is robotically directed to the ROI and it samples all of the cleaved tags. (**E**) The counts are mapped back to tissue location, which produces a spatially resolved digital profile of analyte abundance within each ROI. Abbreviations: UV—ultraviolet; ROI—region of interest.

**Figure 8:**
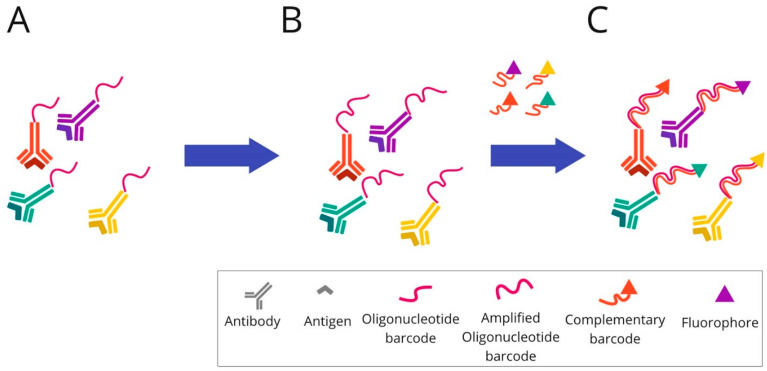
Overview of InSituPlex technique: (**A**) Mixture of target antibodies conjugated with unique oligonucleotide barcodes are used to stain a tissue section. (**B**) Then, the ratio of barcodes per antibody is increased on the tissue through a process that amplifies all targets simultaneously. (**C**) The fluorescently tagged probes complementary to each barcode are added to the sample to hybridise and label each target. Finally, the sections are ready for fluorescence imaging.

Antibodies for individual targets are conjugated with a unique, addressable barcode (DNA oligonucleotide) sequence for consecutive labelling and detection. These techniques include: COdetection by indEXing (CODEX) (Figure 6), Digital Spatial Profiling (DSP) (Figure 7), and InSituPlex (Figure 8).

**CO-detection by indEXing (CODEX)** is a multiplex imaging approach based on DNA barcoding technology and is supported by fluorescent labelling. Contrary to previously described techniques, the primary antibodies are tagged with unique DNA oligonucleotide sequences instead of chromogenic dye, fluorophores, or pure metal chelator labels. In the CODEX method, a cocktail of antibodies is applied to a single-tissue section and imaged in the same instant. Subsequently, the markers are crosslinked to their cellular targets [67]. The procedure of immunostaining is generally performed in one step, and the tissue sections are marked with all the labelled antibodies simultaneously. Up to 60 markers could be visualised and quantified by the CODEX method. This number is limited by recognised DNA barcodes that do not exhibit cross-reactivity with other DNA sequences present in the sample. Visualisation of the antibody attached to the tissue section is possible by adding fluorophores. However, the fluorescent molecule is not linked directly to the antibody–oligonucleotide tag. First, a distinct PCR-based elongation of each DNA oligonucleotide is carried through, followed by complementary DNA strand attachment. A fluorophore is assigned to a complementary oligonucleotide sequence. The analysis is made in cycles. During each imaging cycle, three fluorescently labelled barcodes can be hybridised and give their fluorescent signals. The image is captured, and rehybridisation of complementary DNA strands is conducted using prepared stripping buffer (i.e., H2 buffer mixed with dimethyl sulfoxide—DMSO). The detached sequences and their fluorophores are washed out and the imaging cycle is repeated. Multicycle analysis of 1 cm^2^ area of tissue section takes approximately 30 h at a 400 nm resolution. At the end of the process, the multiparameter image is reconstructed. The CODEX assay platform is commercialised, and the licence belongs to Akoya Biosciences (https://www.akoyabio.com, (accessed on 20 June 2022)). However, fluorescent-signal analysis could be performed by a widely accessible standard fluorescent microscope [67,68].

CODEX multiplexing has recently gained attention in phenotyping immunoregulatory proteins. Its potential in immuno-oncology research was demonstrated by Nolan’s group, who examined human tonsil tissue via a panel of 57 validated antibodies, also covering immune markers [68]. Medrano et al. utilised this technology to describe the spatial arrangement of effector cells and their associated tumour cell targets in relation to each other on sarcoma tissue samples. They evaluated 35 distinct immune markers [69]. The CODEX was also used to characterise melanoma cells’ molecular and spatial features. The study analysed the biopsies of patients receiving anti-PD-1 and CTLA-4 immunotherapies and focused on changes in cellular populations during treatment [70]. The Phillips et al. study provides a complex report covering all the steps (including design, development and optimisation) for the application of a 56-marker antibody panel to cutaneous T-cell lymphoma tissue samples. The whole process could easily be transformed to other neoplasms [71]. The importance of studying cellular composition while preserving spatial information is discussed in the Schürch et al. study, which deeply profiles the immune tumour microenvironment among a cohort of colorectal cancer patients [72].

**Digital Spatial Profiling (DSP)** is a complete commercial system based on the nCounter multiplexing solution, which was designed by NanoString Technologies (the GeoMx™ Digital Spatial Profiling system). It facilitates the high-multiplex spatial profiling of proteins and RNA, creating up to 100-plex and up to 20,000-plex panels, respectively [73]. The principle of the method relies on barcoding technology. Antibodies or RNA hybridisation probes are bound to unique oligonucleotide tags. This connection is made through an intermediary—an ultraviolet (UV) photocleavable (PC) linker—and can easily be lost by using the UV light beam. The possibility of removing the tag using light allows the sample to be reused. After constituting antibodies/RNA probe pairs with photocleavable oligonucleotides (PC-oligos), the tissue is scanned and a digital image is created. The produced image enables the definition of regions of interests (ROIs). Due to precise UV exposure, the oligonucleotide barcodes are liberated and undergo quantitative analysis, followed by mapping back to the tissue location. UV laser light projection is possible due to two digital micromirror devices (DMD), which focus the beam so that the ROI can be directly illuminated. The selected PC-oligo tags are cleaved; then, they are retrieved using a small pipette, sampling 1–2 μL of liquid above each ROI. Subsequently, the collected index barcodes are automatically transported to a multiwell plate and digitally calculated using the NanoString nCounter analysis system. Those digital counts are mapped back to the tissue region representing each ROI, which provides a spatially profiled image of proteins or transcript activity within a distinct tissue sample [74,75,76].

Conventional diagnostic tools are characterised by either limited plex (e.g., IHC) or a lack of spatial resolution (e.g., bulk analysis of nucleic acids) and do not fully depict intratumour heterogeneity. DSP development exceeds these limits and allows for the identification of multiple informative biomarkers and their orientation in a spatial context. The method enables the profiling of up to 800 proteins or mRNA using optical-barcode readout. The hardware limit of spatial resolution is approximately 1 μm^2^, so it is possible to illuminate single T cells (10 μm), which represent the smallest detection target commonly found in immuno-oncology research. Moreover, the system can be automatically configured to multiple modes, such as tumour only; tumour microenvironment only; unique cell types and rare cell features (including PD-L1 expression on macrophages, PD-L1 expression on tumour cells, etc.); spatial gradient around cell features; simple hand-selected geometric areas; or a combination of the above methods [76].

Due to the valuable information obtained using this method, DSP was rapidly adopted in immuno-oncology and tumour-microenvironment research areas. Some examples include immune biomarker evaluation in a cohort of NSCLC [77] or HNSCC tumours from patients receiving immune-checkpoint inhibitor therapy, including PD-1 inhibitors [78]. The DSP technique also turned out to be a helpful tool for immuno-oncology studies in melanoma and allows for the identification of biomarkers that predict responses to immunotherapy [79,80].

It also helps to understand the complex changes in the tumour microenvironment during immunotherapy treatment. The two investigative groups used DSP to characterise the melanoma microenvironment before and during neoadjuvant treatment with an immune-checkpoint inhibitor. Two cohorts were investigated: the first received the PD-1 inhibitor (nivolumab) alone, and in the second, nivolumab was accompanied by ipilimumab, a CTLA-4 inhibitor [81,82].

Recently, more and more research has also been appearing on breast cancer. Some researchers analysed the spatial resolution of tumours or their stromata to identify distinct diversity in immune activation markers [83,84]. The GeoMx Breast Cancer Consortium (GBCC) presented the prospects of the DSP solution in the context of breast cancer microenvironmental investigation. The authors found an application for it in clinical cancer research, whereby it could be adopted to other tumour types characterised by high heterogeneity [85]. The DSP method has also shown its potential in basic biology studies, providing a perspective for future adaptation in the diagnostics of immune markers, including the PD-L1 protein. Gupta et al. used the DSP method in their study to objectively quantified PD-L1 expression in a standardised cell line using an Index tissue microarray [86].

**InSituPlex** technology is also based on DNA barcoding technology. The antibodies for each target are associated with a unique DNA barcode, as in previously described barcoding-based methods. The detection of targets is supported by labelling using fluorescent dye. To spectrally separate the targets during imaging, diverse fluorophores are used.

In the first step, the tissue section is stained using a mixture of primary antibodies conjugated to unique DNA barcodes. Subsequently, the ratio of barcodes per antibody is increased through an amplification technique. The whole process of lengthening the DNA sequence takes place simultaneously for all targets. Next, the complementary probes to each elongated barcode are attached and hybridised. For labelling, all of them are fluorescently marked. Finally, the tissue sections tagged in this way are ready for fluorescence imaging.

InSituPlex is becoming more and more available for immuno-oncology studies. Recently, a commercialised multiplex immunostaining kit (UltiMapper I/O PD-L1) dedicated to this field was developed [87]. InSituPlex seems to be a promising technique for immuno-oncology research. An example of its application in this setting is a study evaluating the phenotype subtypes of immune cells or cell densities among samples of different tumour types such as melanoma, and lung, breast, and colon cancer [88]. In another study InSituPlex was used for the spatial profiling of distinct subpopulations of tumour cells, lymphocytes, and macrophages [89].

The advantages and disadvantages of multiplex imaging technologies described above are summarised in Table 1.

## 3. Conventional IHC vs. Multiplexed Imaging Techniques in PD-L1 Assessment

While conventional IHC is limited by the detection of only one marker at a time, multiplexing techniques could easily visualise multiple targets within a single tissue slide. Thus far, this limitation has not been too problematic in everyday diagnostics. However, in the era of immunotherapy, which is entering the pathological routine, this problem starts to be more and more apparent. The evolution of PD-L1 as a marker of response to immune-checkpoint inhibitors exposes the shortcomings of single-plex assessment. Recent studies have shown that not only is the expression of the marker itself important in predictive assessment, but so its location in the tissue and the type of cell in which it is exposed. Multiplex imaging techniques provide that information. Contrary to conventionally evaluated PD-L1 by IHC, which is subjective and semi-quantitative, multiplexing-based methods provide exact information on which cell in the protein is expressed, which allows for its precise counting, depending on the researcher’s interest [27]. Conventional IHC misses important information about the sample under study, as it is unable to research the co-expression of different markers. However, the assessment of the range of predictive markers together shows its importance in treatment approaches in a variety of tumour types. The presence of PD-L1 on a tumour or in its microenvironment cells is not the only predictive biomarker of a good response for immunotherapy. Tumour-infiltrating lymphocytes (TILSs) are also well-characterised indicators of better treatment results. The presence of TILSs correlates with the expression of PD-L1 in the tumour microenvironment, which implies that patients with neoplasms harbouring both factors should benefit from immunotherapy. Some studies demonstrate that the coexistence of only those two markers has become a predictive tool. That exact interpretation is only possible by using multiplex diagnostic approaches. Even basic chromogenic- or fluorescence-based mIHC/IF methods seems to be sufficient for such an evaluation. Contrarily, in the single-plex analysis, the assessment is roughly estimated by pathologists by sight, resulting in a lack of precision and repeatability [103,104,105,106,107]. Furthermore, there are reports showing that PD-L1 expression is not always associated with an immune infiltrate. This could be an explanation for the significant failure rate of immune-checkpoint inhibitors despite PD-L1 positivity. Researchers have started to look for other predictive factors of good response, such as the spatial distribution of immune markers. The evaluation of PD-L1-positive cells densities, their distribution, as well as their spatial interactions with their microenvironment showed some correlation with a better response to immunotherapy [108]. By using multiplexing, information about the spatial organisation of tested cells is obtained, regardless of the method used. However, in some of the techniques such as MIBI, IMC, or DSP, only ROIs are created, and there are no digital images [61,76]. The other drawback is the elevated sensitivity of cell detection in the tissue sample observed for the TSA method, resulting in some disconcordance compared to testing single markers. Even so, it seems that it does not impact immune-checkpoint inhibitor treatment results; moreover, multiplexing has the strong advantage of predicting the response to immunotherapy. Recent studies revealed that when comparing conventional IHC to mIHC/IF, the degree of diagnostic accuracy in identifying PD-L1 positivity is much higher. Furthermore, the implementation of digital image analysis could improve marker establishment [109]. Recent advances in multiplex-based marker evaluation create a need to develop methods for analysing the data produced. DSP, in particular, needs support from digital imaging programs as it has the potential to generate an enormous number of data, comparable to that obtained by next-generation sequencing (NGS) [76].

## 4. Digital Imaging in Quantitative Pathological Assessment

As the branch of multiplex imaging technology rapidly develops, the need to interpret stained tissue sections is rising. This process is possible thanks to digital pathology (DP), which covers automated image-acquisition (slide scanning) and software-assisted marker visualisation and interpretation. Digital pathology is a multistep process, that includes capturing, storing, and analysing specimens in a digital format. It is of interest as having potential use in everyday practice [110].

### 4.1. Slide Scanners

The basic concept related to digital pathology is whole-slide imaging (WSI), a technique that implies the digitalisation of entire histologic sections, generating “digital slides’’. Capturing WSI has been possible since the 1990s thanks to specialised scanners [111,112,113]. Now, there is a plethora of models commercially available. They vary from high-volume (>100 slides) models—such as Leica (Aperio AT2), Roche (iScan HTo), Olympus (VS120), Philips (UltraFast), Hamamatsu (Nanozoomer S210), 3D Histech (Panoramic 1000), OptraScan (OS-FCL), and Huron (TissueScope LE120)—to low-volume (2−6 slides) scanning systems such as Leica (Aperio LV1), Roche (Ventana DP 200), 3DHistech (Panoramic Desk II), Hamamatsu (Nanozoomer SQ), Sakura (VisionTek M6), Objective Imaging (Glissando), PreciPoint (M8), Mikroscan (SL5), Huron (TissueScope PE), and Motic (EasyScan) [114].

A WSI scanner is an automated microscope that can take a set of photos of an entire glass slide, and by using software, they can be fused into a complex digital image. WSI can be stored in different formats (the most popular are BIF (Ventana), CZI (Zeiss), iSyntax (Philips), NDPI (Hamamatsu), and TIFF), and then, viewed using special software. There is even an option to modify a digital camera to a manual scanner with suitable software [115]. Is it worth mentioning the software tool for reading and writing image data using standardised, open formats over 150 different biological image formats, including many WSI formats [116,117,118]. Some efforts are being made to standardise storage methods and turn digital pathology into a universal method. The Digital Imaging and Communications in Medicine (DICOM) working group’s purpose is to standardise digital imaging across all fields of medicine. One of their efforts focuses on developing open-system scanners, which could store images in a Picture Archiving and Communication System (PACS). The advantage of using PACS for storage is that it utilises DICOM-standard communication. DICOM is widely implemented in electronic medical-record systems based on imaging and has become the standard for imaging in medicine worldwide [86]. Moreover, digital-pathology vendors such as Roche-Ventana, Leica-Aperio, and Philips have already integrated the DICOM standard file format and network protocol into their products [119,120,121,122].

However, this format is still not widely adopted for WSI, and file converters are necessary. Recently Gu et al. presented dicom_wsi, which is a toolkit based on the Python programming language that enables the conversion of WSI to common DICOM. However, the image should be saved in a format supported by OpenSlide, a C library that supplies an interface for reading WSIs and assisting with variable vendor solutions (Ventana (.bif, .tif), Hamamatsu (.vms, .vmu, .ndpi), Philips (.tiff) Lecia (.scn), MIRAX (.mrxs), Generic tiled TIFF (.tif), Trestle (.tif), Sakura (.svslide), and Aperio (.svs, .tif) [115].

Thus far, there are no universal standards by which scanners can be compared to each other; however, there are a few parameters that can be considered: imaging (brightfield vs. fluorescence); scan time (time/slide (ex: 60 s)); throughput (slides/time (ex: 30 slides/h)); magnification: enlargement to sensor (it is worth mentioning that objective microscope lenses of 20×–60× are “air”, but 80×–100× lenses needs oil immersion); resolution: smallest identifiable distinguished detail (resolution is a combination of optical magnification, net optical aperture, and sensor resolution); loading mechanisms (automated/manual); total capacity (maximum unattended operation); cartridge size (number of slides per load unit); continuity (ability to load without interruption); scan types (single-plane vs. Z-stacking); and slide format: 1 × 3 (25 × 75 mm) or larger. The major problems with WSI are concerned with large files which require additional time to load and manipulate over a slow network connection; Z-stack and high magnification are especially challenging. A slower reaction time makes the use of DP in cytological and haematological material less efficient and somewhat problematic. Additionally, the retention of WSI over time will require abundant storage space [123,124]. Some systems such as Vectra-Polaris™/InForm Cell Analysis/Akoya/PerkinElmer [25], MultiOmyx/analysis software [125], and Aperio FL/digital image-analysis tools [126] are becoming more and more popular in multiplex digital imaging. Their main advantage is combining image-analysis systems with automated scanning [99].

### 4.2. Open-Source Software

Appropriate software is needed to analyse complex images created using various IHC techniques. Such tools are able to develop algorithms that support the visualisation of results and help to compare different images and indefinite specific tissue or cell types. Those programs are commercially available or are open-source. Usually, open-source software is more complicated at the beginning of usage. Still, some professional online forums exist (i.e., https://forum.image.sc/, (accessed on 20 June 2022) or https://github.com/, (accessed on 20 June 2022)), which help find solutions in many cases and are more flexible to use.

**Qupath** (https://qupath.github.io, (accessed on 20 June 2022)) is one of the open-source programs created by Dr. Pete Bankhead at Queen’s University Belfast. It is a user-friendly interface specially designed to analyse WSI in both fluorescence and brightfield technology. Initially, its application was mainly focused on IHC quantification analysis. However, later, a lot of additional options for tumour spatial analysis on hematoxylin and eosin slides were added. One of the most significant advantages of this program is its ability to operate on whole slides—a tremendous number of data. Moreover, it comprises a user-friendly WSI viewer with easy-to-use annotation tools and expanded visualisation tools. The instruments for making annotations are very helpful, making the workflow smoother. The pixel information is used for boosting the precision and speeding up the process of annotations. For example, the semi-automatic separation of two tissue compartments based on colour contrast is possible. QuPath provides both ready-to-use as well as customised solutions. The artificial intelligence (AI) algorithms offered by the program are sufficient to resolve most popular problems concerning the automatically driven detection and classification of particular cells, etc. The created workflows can be flexibly changed. It usually consists of steps such as: (1) building a multi-slide project supported by automated tissue-detection and stain estimation; (2) detecting cells and measuring them; (3) creating classifiers for markers—set parameters of annotated objects or pixel values that are helpful for the user; and (4) combining the last two steps and applying them to the cells on the whole slide or part of the slide. Qupath provides the possibility to copy one’s workflow to create a script (an automated sets of workflows). Those scripts can be further used for other formats. The images and measurements created in Qupath can be extracted to other external software applications, with extraction to ImageJ being particularly easy. The data then can be applied in further research through batch processing and survival analysis. It is worth noting that this software is still actively being developed. Upgrades are mostly conducted for new tools, a better, user-friendly interface, and better support for immunofluorescence imaging analysis [127,128].

**ImageJ** (previously NIH Image) (https://imagej.nih.gov/ij/index.html, (accessed on 20 June 2022)) is an image-processing program developed by the National Institutes of Health (NIH) and the Laboratory for Optical and Computational Instrumentation (LOCI) at the University of Wisconsin as a collaboration project. Because of its popularity and wide adoption, a lot of plugins have been developed. Those plugins can be useful for various very specific tasks in microscopy analyses. ImageJ is compatible with Bio-Formats. Unfortunately, when it comes to WSI, the size of an image is an issue, because ImageJ cannot handle such large files. To solve this issue, new plugins were established (the Qupath software started as an ImageJ plugin) [129,130,131,132,133].

**Cell Profiler** [www.cellprofiler.org, (accessed on 20 June 2022)] was developed by the Broad Institute of MIT and Harvard. It is an open-source, free to use software package that facilitates the analysis of cells in distinct image formats [131]. The adjustable design of Cell Profiler permits the user to point and click to accomplish most tasks. Analysis is object-based, which means that it determines the area of images and evaluates criteria such as size and shape. The objects are then detected and generated hierarchically (primary objects are usually cell nuclei); later, those objects serve as a base for secondary objects. Those secondary objects are cells which consist of the previously identified nuclei (primary objects) and their neighbourhoods. To create secondary objects, the software measures features such as the area, colour intensity, degree of correlation between colours, shape, texture (smoothness), and number of neighbours. The shapes of the created objects are shown as a mask on the original image. Such a display allows for verification and quality control. Similarly to the Qupath program, the individual processes can be combined into a “pipeline”, which can then be used for further automatic analyses of images. An exemplary “pipeline” consists of steps similar to those used in Qupath: loading the images, adjusting for uneven illumination, recognising the objects, and taking measurements of the objects. These modules can be freely rearranged within a pipeline. The software is not suitable for whole-slide imaging; however, it is possible when integrated with other software [134,135,136].

**Icy** (https://icy.bioimageanalysis.org, (accessed on 20 June 2022)) is another free, open-source software package. It was founded by the Institut Pasteur, BioImaging, France. Its creators describe it as “a collaborative photoshop dedicated to image analysis”. Users are able to visualise, annotate and quantify data from images. This software also manages to evaluate whole slides. The design of Icy is grounded in components to which the user is already adjusted. The main ‘ribbon’ toolbar is similar to Office Suite by Microsoft, and it grants access to all the software options. The Icy software was designed both for image-analysis scientists, to facilitate the development of new algorithms, and for life-science scientists, to provide instinctive image-analysis tools [137,138]. The most frequently used software packages are summarised in Table 2.

### 4.3. Artificial Intelligence (AI)

In the last few years, there has been a lot of interest in using AI and machine learning (ML) for analysing data gained via DP. Very often, such studies are interested in comparing AI with pathologists and trying to provide repeatable results in identifying various parameters of tumours. The potential benefits are more repeatable cell counting and measurement, and saving pathologists time in their everyday work routine [27]. Additionally, by measuring many cell and inter-cell parameters, AI can potentially help find new HE phenomena that can later be used [140,141,142]. Some commercially available software is made for breast cancers and scoring ER, PGR, Ki67, and HER-2. Free open-source software allows for even faster research in digital pathology [128].

The treatment of advanced NSCLC has been dramatically improved thanks to immunotherapy. PD-1 and PD-L1 inhibitors are used in this type of cancer with success. The scoring of PD-L1 using IHC staining is often used to predict the likelihood of responses to therapy [27,143,144]. There are three widely known PD-L1 scoring methods: (1) the tumour proportion score (TPS), which is defined by the percentage of all tumour cells presenting membranous PD-L1 expression; (2) the combined positive score (CPS), which is a sum of the positively stained tumour cells and infiltrating immune cells and is divided by the total number of viable tumour cells, and then, multiplied by 100; and (3) the tumour-infiltrating immune cell score (ICS), which illustrates the percentage of the area of PD-L1-positive infiltrating immune cells with respect to the whole tissue area of the tumour slide [145]. As with many IHC-based scoring systems, there is variability between pathologists [27,143]. This is a challenging task due to: poorly circumscribed and/or heterogeneous tumours; the intra-tumoural heterogeneity of PD-L1; and pre-analytical variables that affect tissue such as fixation time, endogenous and exogenous pigments within the tissue, staining expression in other tissue compartments (stromal and inflammatory cells), and misinterpretation in IHC evaluation (staining of necrosis or cytoplasmic staining artefact) [144,146]. Many studies show that the use of AI and DP in PDL1 are comparable with pathologist scoring. A comparison of different methods of DP and AI-assisted studies is shown in Table 3.

## 5. Conclusions

In the era of immunotherapy, there is an urgent need for multiplexed imaging methods to be routinely used in cancer diagnostics. The complexity of studying the tumour-microenvironment demands new techniques that allow for more sophisticated analysis of immune cell phenotypes, their spatial patterns, and their interactions with each other and with cancer cells. Predicting a patient’s likelihood to respond to a particular therapy seems to be the main issue to solve in the near future. Multiplex labelling could help to stratify patients for appropriate immune treatment and determine candidates who would benefit from immunotherapy. It seems to use the potential of this therapy as effectively as possible without exposing non-responders to unnecessary side-effects and costs. The different multiplexed technologies described in this review have shown their utility in tumour-microenvironment research and translational studies, which could be applied in cancer diagnostics. However, developing these new methods requires standardisation, optimisation, and validation for specific cancer types. For further implementation in clinics to become possible, multicentre studies need to be performed. Moreover, multidisciplinary teams, including pathologists, oncologists, immunologists, bioinformaticians, and engineers, should be established to most effectively and fully develop this promising quantitative approach to cancer immunotherapy.

## Figures and Tables

**Table 1:** Summary of the advantages and disadvantages of multiplex imaging technologies based on [27,44,59,61,68,76,90,91,92,93,94,95,96,97,98,99,100,101,102].

Method	Advantages	Disadvantages
mIHC	Low cost and automation of staining. The simplicity of usage and interpretation. Established guidelines and protocols. Standard light microscope for interpretation.	Co-expression studies require careful selection of the chromogen pairs, and due to the limited amount of tissue on one slide, only a restricted number of chromogens can be used. Semiquantitative method, unable to assess marker intensity.
MICSSS	It is a simple and relatively affordable technique, similar to standard chromogenic immunohistochemistry. Ability to preview the entire slide for each marker. Each marker is individually stained, excluding staining or signal interferences. Standard light microscope for interpretation.	Time-consuming method due to slow throughput. It allows the marking of up to 10 biomarkers on a single slide for 10 days (6 h per cycle). Possibility of mechanical tissue damage and formation of artefacts during the coverslip removal procedures. Difficulties with coregistration of images on the the whole slide due to their large number and complicated software service.
TSA	It allows spatial-arrangement analysis of multiple targets within a single tissue section. Any primary antibody validated for IHC, regardless of host species, can be used for each target of interest. The autofluorescence can be rectified by a multispectral microscope. Purified fluorophores are commercially accessible. When compared to chromogenic-based methods, multiplex immunofluorescence has a larger linear dynamic range, which makes it easier to study the marker intensity. Costs are comparable to standard chromogenic-based methods.	There is an elevated risk of human-error occurrence, while manual staging is difficult. However, the use of autostainers could help to overcome the problem. There is a risk of “fluorophore bleed-through” or “umbrella effect” due to excessive tyramide deposition. Spectral overlap is a problem when the above seven probes are analysed.
IMC	Absence of tissue background signal. Highly quantitative method due to the absence of matrix effects. No need for serial slides to raise the target number or cyclic rounds of labelling–stripping–acquisition of the same tissue section. Up to 40 markers on an individual tissue section at a single-cell level can be analysed. The information on tissue architecture and cellular morphology is preserved. Markers can be analysed in parallel for a single section of tissue with low channel crosstalk.	When compared to fluorescence imaging methods, the subcellular resolution is diminished. Laser-ablated tissue is not reusable for subsequent applications. More expensive than techniques based on fluorophore-conjugated antibodies. Advanced analysis tools are required. Increasing the processing speed is limited in this method. The main limitation is the risk of cross-contamination between laser-ablation spots. The analysed slide is not imaged. Because of the time required to perform ablation, ROI size is limited. Lower sensitivity than fluorescence imaging techniques as it lacks signal amplification or possibility to raise exposure time.
MIBI	Absence of tissue background signal. Quantitative information can be obtained from the types of cells and their distribution within the tissue. Markers can be analysed in parallel for a single section of tissue with low channel crosstalk. Image resolution, as well as depth of sample acquisition, can be adjusted. Has the capability of reaching sensitivity as low as parts-per-billion with a dynamic range of 10^5, and preserves very high resolution. It is capable of analysing up to 100 markers on a unique tissue section	More expensive than techniques based on fluorophore-conjugated antibodies. The entire tissue slide is not a digital image; it is only the ROI.
CODEX	Can simultaneously reveal up to 60 markers in an individual tissue section. Lack of cross-reactivity (oligonucleotide–oligonucleotide, tissue or cellular DNA). It provides information about biomarkers’ relative number and expression at a spatial level. Relatively cost-effective and quick method.	It lacks a signal-amplification system. Baseline autofluorescence of tissues present. Unified staining protocol demands that each antibody be individually conjugated and validated. The antibodies used in the CODEX system are expensive.
DSP	Simultaneous measurement of all markers. Possibility to create up to an 800-plex assay. However, when applying the NGS readout mode, the multiplexing is unlimited. Repeated cycles of high-plex profiling or subsequent DNA sequencing on the same tissue section are available. No autofluorescence is present.	No single-cell expression data. Profiling every cell in a tissue slice at single-cell resolution is costly and tedious. It cannot create an image.
InSituPlex	More reproducible than other multiplexing techniques. Lower complexity of the laboratory test, fewer component reagents to prepare, fewer retrieval steps required, automated staining run, and no need for complex prevalidation when compared to multiplex-fluorescence techniques. An assay protocol can be easily implemented in laboratories with the standard fluorescent microscope, because it is compatible with standard IHC workflows and automation instrumentation. It preserves the integrity of the tissue sample.	A small number of publications are available.

**Table 2:** The characteristics of the most popular software used for image analysis based on [127,132,135,138,139].

	QuPath	ImageJ	CellProfiler	Icy
Type of imaging	Brightfield and fluorescence	Brightfield and fluorescence	Flow cytometry, brightfield, darkfield, or fluorescence	Brightfield and fluorescence
Handle to WSI	Yes	No (needs plugin)	No (needs other programs)	Yes
IHC analysis	Yes	Yes	Yes	Yes
Bio-format	Yes	Yes (with plugin)	Yes	Yes
Other advantages	Built-in cell segmentation and classification software, pixel clarifier, smart annotation tools	Many plugins developed	The user-friendly interface supports 3D images	Supports 3D images, tracking moving cells
Disadvantages	Some options require programming skills to use	Some plugins need programming skills to use	Small number of plugins or plugins that overlap in their functionality.	Designed for researchers with software-development skills

**Table 3:** Overview of AI-assisted methods of digital pathology for PD-L1 assessment.

Ref.	[147]	[148]	[145]	[143]	[127]
Aim of the study	PD-L1 expression evaluation using digital-image analyses correlated with pathologist interpretation.	Domain adaptation-based deep learning for automated tumour-cell scoring on PD-L1 stained tissue sections.	Automated PD-L1 scoring applying artificial intelligence.	Automated PD-L1 scoring applying open-source software.	QuPath performance testing.
Type of cancer	Gastric cancer	Non-small-cell lung cancer	Head and neck squamous cell carcinoma	Non-small-cell lung cancer	Colorectal cancer
Method	IHC	IHC	IHC	IHC	IHC
Tools	FDA-cleared Aperio Imagescope IHC Membrane Image-Analysis software (ScanScope, Aperio Technologies, Vista, CA, USA)	Deep-learning-based image- analysis software (DASGAN network)	QuPath	QuPath	QuPath
Conclusions	No significant difference in interpretation between pathologist and digital analysis	Software replicates the pathologist’s assessment	Comparable results between human-to-human and human-to-AI interpretation.	Similar interpretation between pathologist and digital analysis	There is incipient evidence that software helps in investigating PD-L1 prognostic value in colorectal cancer
